# Profiling of fatty acid metabolism in the dorsal root ganglion after peripheral nerve injury

**DOI:** 10.3389/fpain.2022.948689

**Published:** 2022-07-29

**Authors:** Shota Yamamoto, Tomomi Hashidate-Yoshida, Takao Shimizu, Hideo Shindou

**Affiliations:** ^1^Department of Lipid Life Science, National Center for Global Health and Medicine, Tokyo, Japan; ^2^Institute of Microbial Chemistry, Tokyo, Japan; ^3^Department of Lipid Signaling, National Center for Global Health and Medicine, Tokyo, Japan; ^4^Department of Medical Lipid Science, Graduate School of Medicine, The University of Tokyo, Tokyo, Japan

**Keywords:** dorsal root ganglion, fatty acid, leukotrien, lipid mediator, pain, peripheral nerve injury, prostaglandin

## Abstract

Peripheral nerve injury (PNI) induces neuronal hyperexcitability, which underlies neuropathic pain. The emergence of RNA sequencing technologies has enabled profiling of transcriptional changes in pathological conditions. However, these approaches do not provide information regarding metabolites such as lipids that are not directly encoded by genes. Fatty acids (FAs) are some of the essential lipids in mammalian organisms and are mainly stored as membrane phospholipids. In response to various biological stimuli, FAs are rapidly released and converted into several mediators, such as eicosanoids and docosanoids. FAs themselves or their metabolites play important roles in physiology and pathology. In this study, using a comprehensive lipidomic analysis of FA metabolites, 152 species were measured in the dorsal root ganglia of mice at multiple time points after PNI. We found that PNI increased the ω-6 FA metabolites produced by cyclooxygenases but not those produced by lipoxygenases or cytochrome P450 enzymes in the dorsal root ganglia. In contrast, ω-3 FA metabolites biosynthesized by any enzyme transiently increased after nerve injury. Overall, these findings provide a new resource and valuable insights into PNI pathologies, including pain and nerve regeneration.

## Introduction

Peripheral nerve injury (PNI) induces refractory pain syndrome, which is known as neuropathic pain. Neuropathic pain is often caused by damage to the nervous system resulting from diabetes, cancer, chemotherapy, viral infection, autoimmune disease, stroke, and trauma (1). Although neuropathic pain is estimated to affect 7–10% of the human population (2), there is currently no effective treatment. However, advances in understanding the pathology of neuropathic pain have revealed the potential for novel therapeutic strategies (3).

Recently, the emergence of RNA sequencing techniques and relevant technological improvements have greatly advanced life science research, including pain research (4–6). However, these approaches cannot provide information regarding small-molecule metabolites that are not directly encoded by genes. Among metabolites, lipids play pivotal roles in physiological and pathological conditions (7, 8). In particular, fatty acids (FAs) have diverse roles in mammalian organisms, and most of them are stored as glycerophospholipids (9) [side note, FAs can be illustrated as XX:Yω-Z (where XX, Y, and Z are carbon number, double bond number, and the position of the carbon having the first double bond from the methyl-end, respectively)]. Various biological stimuli can induce the release of FAs through phospholipase A_1/2_ reactions. These FAs can be further metabolized into diverse lipid mediators by three major enzymes, cyclooxygenases (COX1 and 2), lipoxygenases (LOX), and cytochrome P450 (CYP) enzymes (10). Lipid mediators exert their biological functions through G-protein coupled receptors, ion channels, and possibly nuclear receptors (10, 11).

Accumulating evidence has gradually clarified the role of lipid mediators in the pathophysiology of pain (12–14). For example, prostaglandin E_2_ (PGE_2_) and PGI_2_, which are COX-produced metabolites of arachidonic acid (AA, 20:4ω-6), induce sensitization of transient receptor potential vanilloid 1 (TRPV1) *via* intracellular protein kinase and are involved in inflammatory pain (15, 16). Moreover, 9-hydroxyoctadecadienoic acid (9-HODE) and 13-HODE, which are LOX-produced metabolites of linoleic acid (LA, 18:2ω-6), could also activate TRPV1 and cause inflammation-induced pain hypersensitivity (17, 18). In contrast to the roles of ω-6 FA in promoting pain, ω-3 FA metabolites, such as eicosapentaenoic acid (EPA, 20:5ω-3) and docosahexaenoic acid (DHA, 22:6ω-3), are reported to improve pain. These ω-3 FA-derived anti-inflammatory lipid mediators are coined “specialized pro-resolving lipid mediators” (SPMs) (19, 20). Recently, there have been increasing reports regarding the pharmacological efficacy of SPMs in the treatment of pain disorders (21–23), although the biosynthetic pathways, receptors, and *in vivo* presence of SPMs remain to be validated (24). Whereas previous studies have reported the pathophysiological roles of specific lipid mediators of interest, comprehensive lipidomic information on pain pathology is currently lacking.

To address this gap, we used a PNI-induced neuropathic pain model to conduct an extensive lipidomic analysis of FA metabolites in the dorsal root ganglion (DRG), the cell body assembly tissue of primary afferents. Moreover, we monitored the quantitative changes in 152 species of FA metabolites in the DRG after PNI over time, focusing on the metabolites of ω-6 FA, such as AA and LA.

## Results

**Figure 1:**
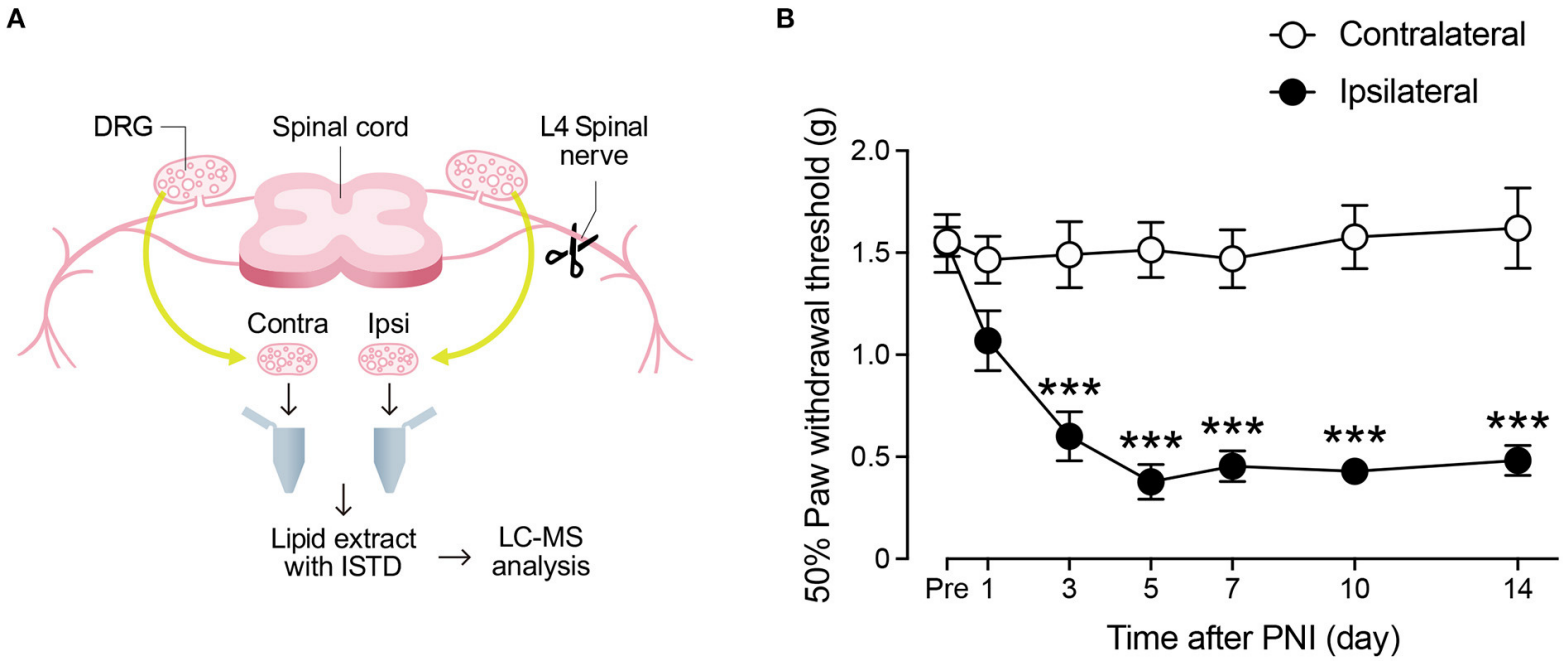
Schematic diagram of lipidomic analysis. **(A)** Schematic diagram of the experimental protocol for measuring fatty acid metabolites (Contra, contralateral; Ipsi, ipsilateral; ISTD, internal standard). See also Material and Methods. **(B)** Paw withdrawal threshold to mechanical stimuli before and after peripheral nerve injury (PNI) (*n* = 8; ***P < 0.001 vs. the contralateral side). Values are means ± s.e.m.

We performed lipidomic analysis of the lumbar DRG from mice after PNI (Figure 1A), over multiple time points ranging from 1 to 14 days after injury. First, we observed that PNI induced mechanical allodynia from day 3, which was suspended until day 14 (Figure 1B). Next, we monitored 152 species of FA metabolites and free FAs (AA, EPA, and DHA) using liquid chromatography-tandem mass spectrometry (LC-MS/MS), and used standard lipids to create a standard curve for data qualification and quantification (Supplementary Table 1). We analyzed pooled L4 DRG samples from 6 to 8 mice combined because we could constantly detect only approximately 10 species within the calibration range from the DRG tissue of one mouse. In the present study, 44 species were detected within the quantifiable range in most samples. We classified each metabolite by its synthetic pathway according to a previous study (25), even though some FA metabolites can be synthesized through more than 2 pathways. The results were expressed as the average of the quantitative values for each lipid molecule per DRG tissue (picogram/DRG), and the raw data are listed in Supplementary Table 2.

### COX-produced metabolites are increased from 7 days after PNI

**Figure 2:**
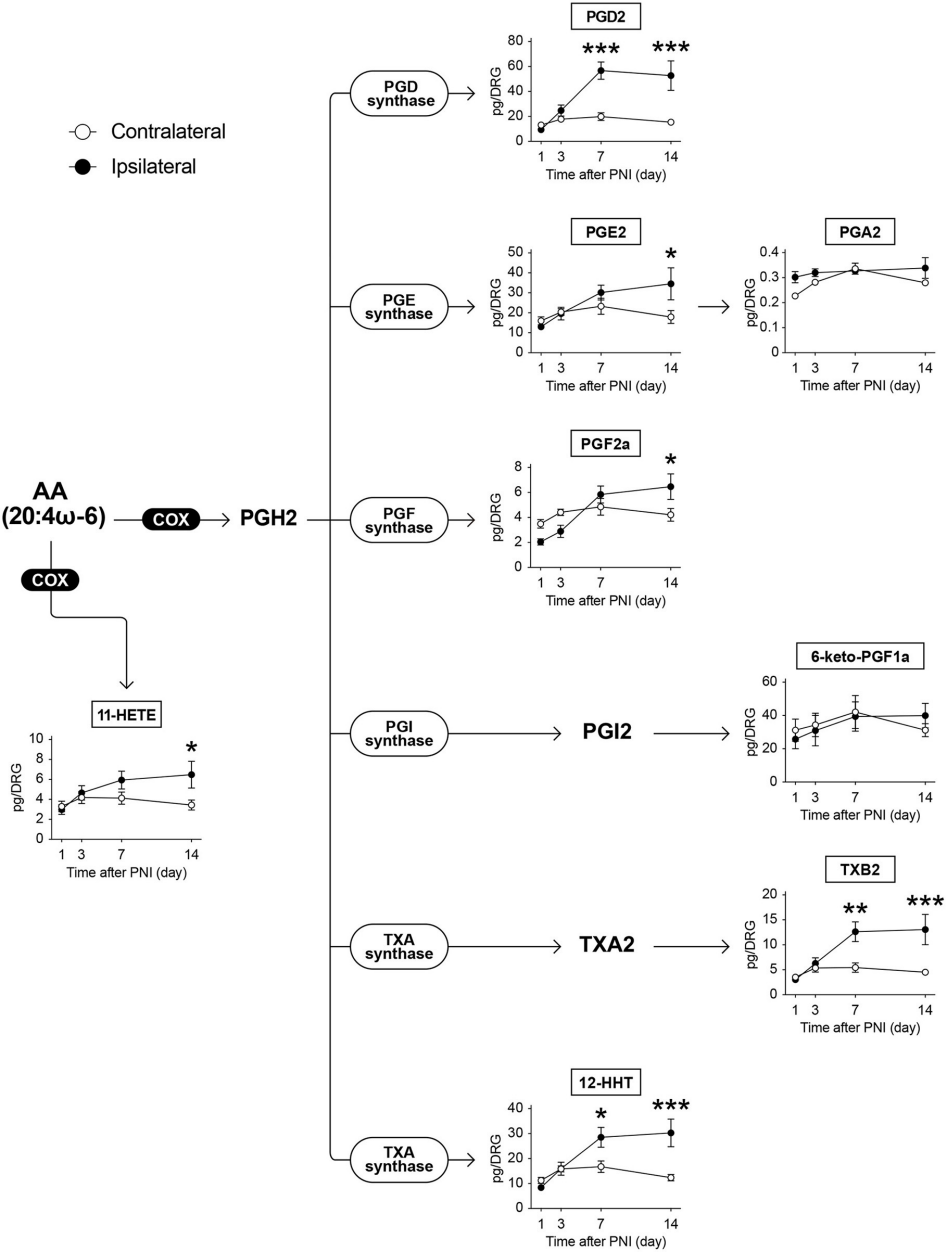
PNI increases AA-derived COX-produced metabolites in the DRG. The metabolic pathways of arachidonic acid (AA) after oxidation by cyclooxygenase (COX) enzymes; eight species could be quantified (*n* = 3–6; *P < 0.05, **P < 0.01, ***P < 0.001 vs. the contralateral side). Values are means ± s.e.m. The presented data are from one set of experiments (PGA_2_), or compiled from two sets of experiments (others). The raw data are listed in Supplementary Table 2.

AA can be subjected to COX-mediated oxidization and is metabolized to PGH_2_, which is further metabolized to prostanoids, such as PGD_2_, PGE_2_, PGI_2_, and thromboxane A_2_ (TXA_2_), by terminal PG synthases (Figure 2). Among the 152 species of FA metabolites measured, we detected eight species of COX-produced AA metabolites: PGA_2_, PGD_2_, PGE_2_, PGF_2α_, 6-keto-PGF_1α_ (a metabolite of PGI_2_), TXB_2_ (a metabolite of TXA_2_), 12-hydroxyheptadecatrienoic acid (12-HHT), and 11-hydroxyeicosatetraenoic acid (11-HETE) (Figure 2). We found that PNI did not alter the levels of these metabolites on days 1 and 3 after PNI, despite the detection of pain behavior on day 3. However, most of their levels, except those of PGA_2_ and 6-keto-PGF_1α_, gradually increased and remained constant until day 14. In addition, 11-HETE also showed the same alteration pattern as the downstream species of PGH_2_, even though it was not biosynthesized *via* PGH_2_. The levels of PGA_2_ and 6-keto-PGF_1α_ were not changed by nerve injury (Figure 2). Furthermore, PNI did not alter the level of free AA in the DRG (Supplementary Figure 1A).

**Figure 3:**
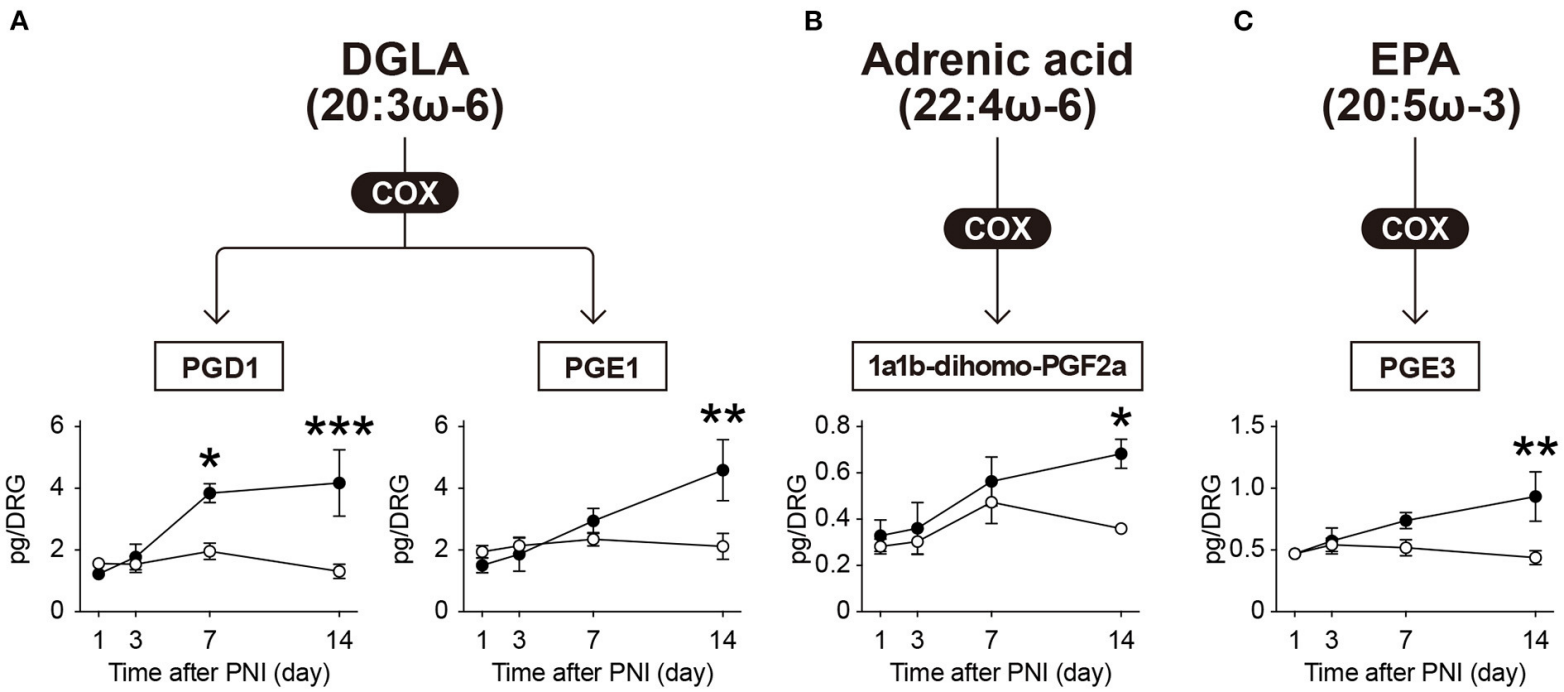
The levels of COX-produced metabolites (other than those derived from arachidonic acid) are elevated after PNI. The metabolic pathways of **(A)** dihomo-γ-linolenic acid (DGLA), **(B)** adrenic acid, and **(C)** eicosapentaenoic acid (EPA) after oxidation by cyclooxygenase (COX) enzymes; a total of four species could be quantified (*n* = 4–6; *P < 0.05, **P < 0.01, ***P < 0.001 *vs*. the contralateral side). Values are means ± s.e.m. The presented data are compiled from two sets of experiments. The raw data are listed in Supplementary Table 2.

In addition to the above-mentioned species, we detected PGD_1_ and PGE_1_ (metabolites of dihomo-γ-linolenic acid, 20:3ω-6), 1a,1b-dihomo-PGF_2α_ (a metabolite of adrenic acid, 22:4ω-6), and PGE_3_ (a metabolite of EPA) (Figure 3). We found that these metabolites were increased in the same manner as AA metabolites after PNI (Figure 3). Although we also found that PNI increased the level of free EPA (Supplementary Figure 1B), which was not reflected in the level of PGE_3_. These results suggest that PNI increases most of the COX-produced lipid mediators in the DRG, regardless of whether they are derived from ω-6 or ω-3 FA, and that these increases correlate temporally with the development of mechanical allodynia from day 7 onward.

### PNI increases LOX-produced metabolites derived from ω-3 FA but not from ω-6 FA

**Figure 4:**
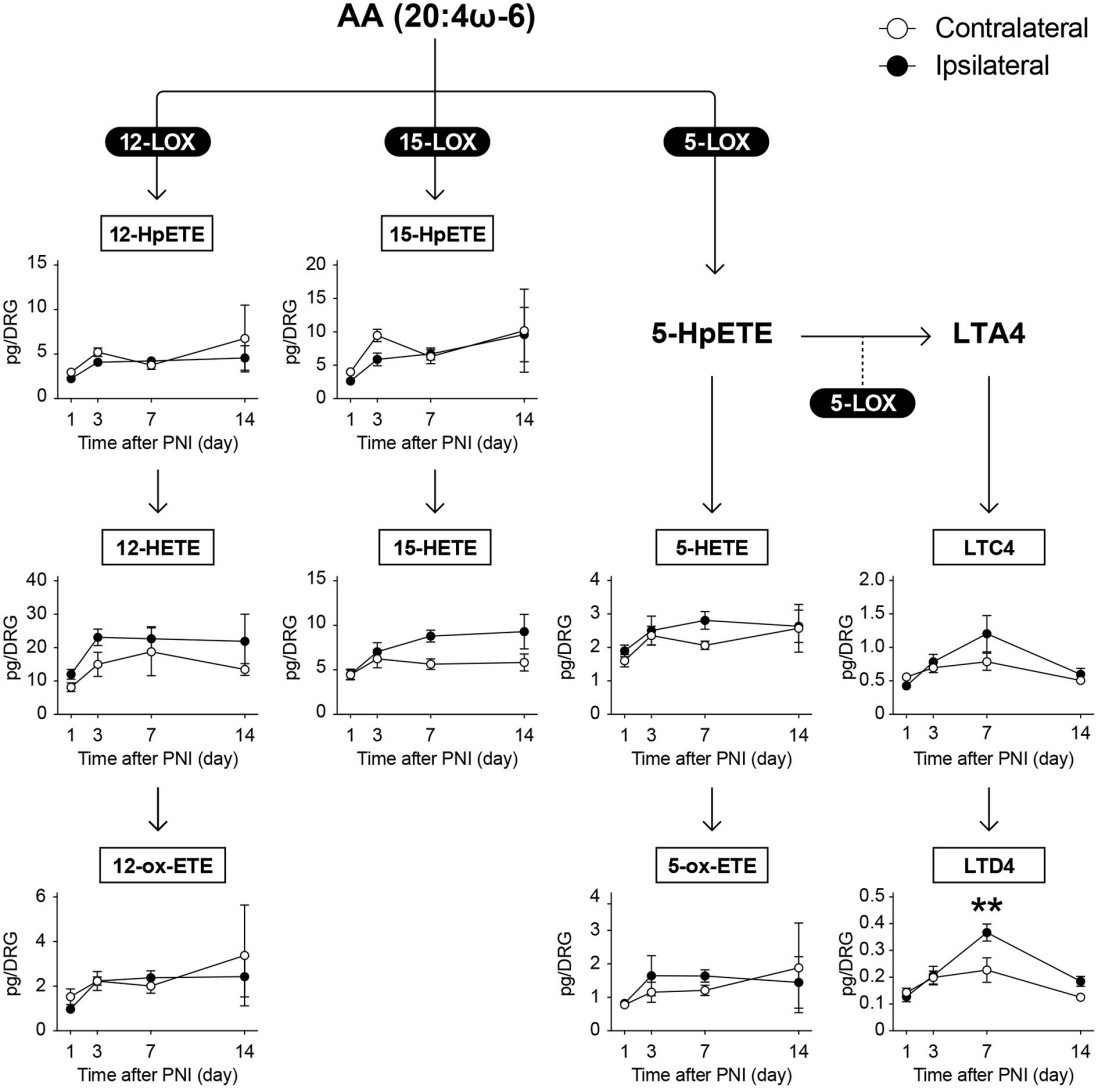
PNI does not affect most of the AA-derived LOX-produced metabolites in the DRG. The metabolic pathways of arachidonic acid (AA) after oxidation by lipoxygenase (LOX) enzymes; nine species could be quantified (*n* = 3–6; **P < 0.01 vs. the contralateral side). Values are means ± s.e.m. The presented data are from one set of experiments (5-ox-ETE, 12-HpETE, 12-ox-ETE, and 15-HpETE), or compiled from two sets of experiments (others). The raw data are listed in Supplementary Table 2.

There are several subtypes of LOX enzymes including 5-LOX, 12-LOXs, and 15-LOX. When AA is metabolized by 5-LOX, 5-hydroperoxyeicosatetraenoic acid (5-HpETE) is produced, which is further metabolized to leukotriene A_4_ (LTA_4_) *via* 5-LOX-mediated dehydration. In contrast, HETEs are produced when HpETEs are subjected to reaction with peroxidase, and further oxidation produces ox-ETEs (also known as KETEs) (Figure 4). In our measurement, we detected 16 and 5 species of LOX-produced metabolites derived from ω-6 and ω-3 FA, respectively.

**Figure 5:**
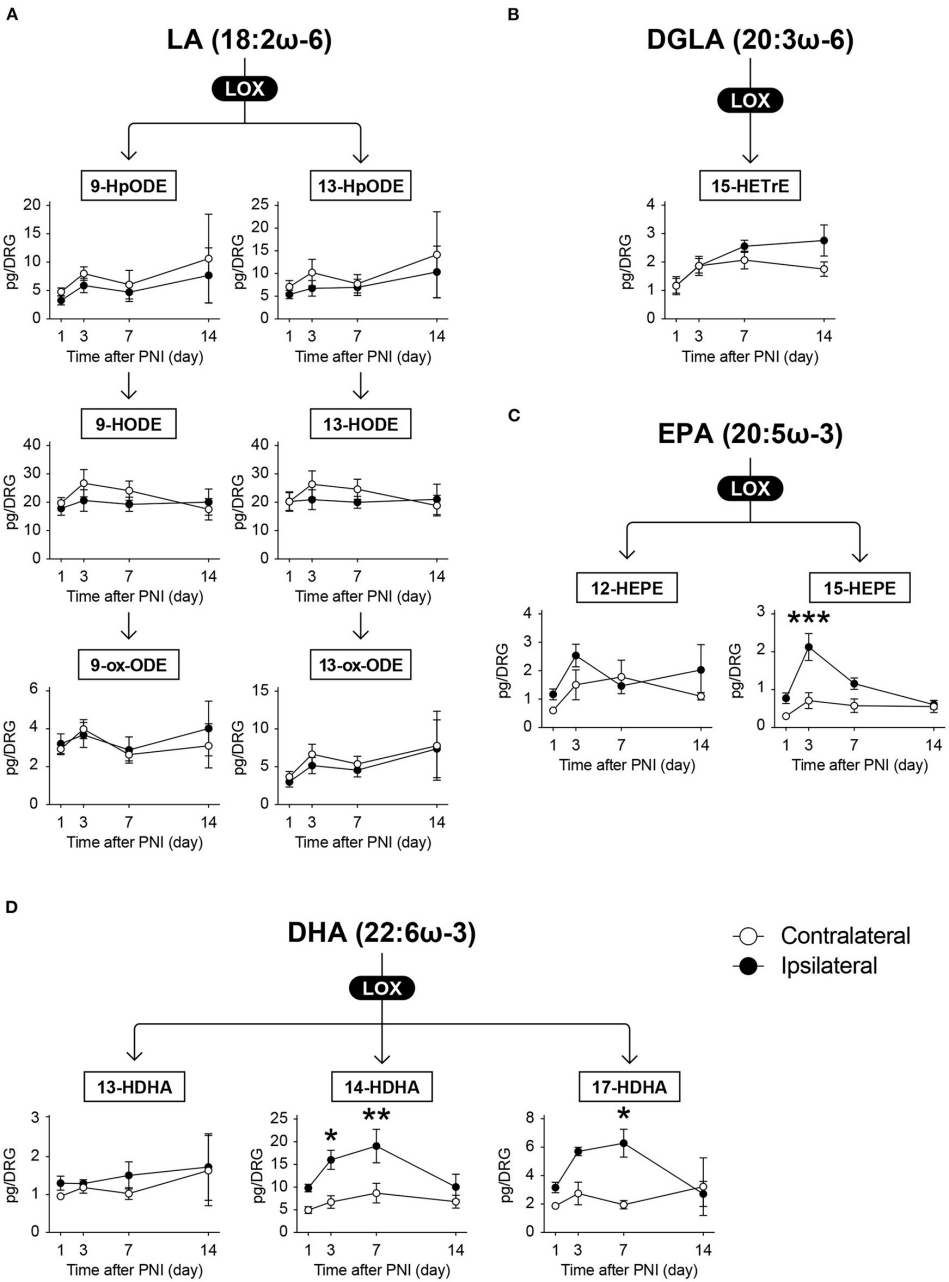
Omega 3 FA-derived metabolites synthesized by LOX enzymes are transiently increased after PNI. The metabolic pathways of **(A)** linoleic acid (LA), **(B)** dihomo-γ-linolenic acid (DGLA), **(C)** eicosapentaenoic acid (EPA), and **(D)** docosahexaenoic acid (DHA) after oxidation by lipoxygenase (LOX) enzymes. In total, six and five species could be quantified from ω-6 and ω-3 fatty acids (FAs), respectively (*n* = 3–6; *P < 0.05, **P < 0.01, ***P < 0.001 vs. the contralateral side). Values are means ± s.e.m. The presented data are from one set of experiments (9-HpODE, Day 1 of 13-HpODE, 15-HEPE, 13-HDHA, and 17-HDHA), or compiled from two sets of experiments (others). The raw data are listed in Supplementary Table 2.

We detected LTC_4_, LTD_4_, 5-HETE, 5-ox-ETE, 12-HpETE, 12-HETE, 12-ox-ETE, 15-HpETE, and 15-HETE as AA metabolites (Figure 4); 9-hydroperoxyoctadecadienoic acid (9-HpODE), 9-HODE, 9-ox-ODE (also known as 9-KODE), 13-HpODE, 13-HODE, and 13-ox-ODE as LA metabolites (Figure 5A); and 15-hydroxytrienoic acid (15-HETrE) as a metabolite of dihomo-γ-linolenic acid (Figure 5B). We found that PNI significantly increased the level of LTD_4_ on day 7 (Figure 4). Although some other species showed an increasing trend (15-HETE, *P* = 0.0827 on day 14; LTC_4_, *P* = 0.0848 on day 7), the amounts of other LOX-produced metabolites were not altered after nerve injury (Figures 4, 5A,B). Regarding ω-3 FA, we detected 12-hydroxy-EPA (12-HEPE) and 15-HEPE as EPA metabolites (Figure 5C), and 13-hydroxy-DHA (13-HDHA), 14-HDHA, and 17-HDHA as DHA metabolites (Figure 5D). In contrast to the metabolites from ω-6 FA, LOX-produced metabolites from ω-3 FA were significantly increased after PNI. Moreover, these species were increased from day 3 to 7 after nerve injury and returned to levels comparable with those of the contralateral side by day 14 (Figures 5C,D). Furthermore, these metabolites did not correlate with the amount of free fatty acids (Supplementary Figures 1B,C). These results indicate that LOX-produced metabolites derived from EPA and DHA in the DRG are lipid species that transiently increase during the onset of mechanical allodynia.

### CYP-produced metabolites show changes similar to LOX-produced metabolites

**Figure 6:**
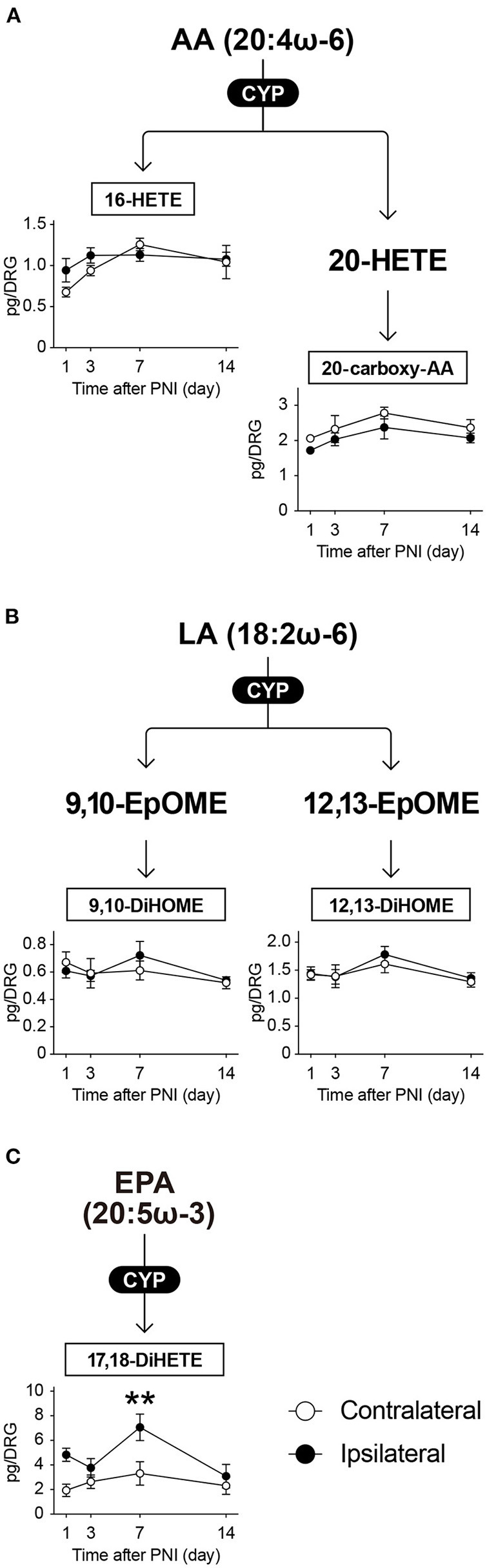
CYP-produced EPA metabolite 17,18-DiHETE is transiently increased after PNI. The metabolic pathways of **(A)** arachidonic acid (AA), **(B)** linoleic acid (LA), and **(C)** eicosapentaenoic acid (EPA) after oxidation by cytochrome-P_450_ (CYP) enzymes. In total, five species could be quantified (*n* = 3–6; **P < 0.01 *vs*. the contralateral side). Values are means ± s.e.m. The presented data are from one set of experiments (16-HETE and 20-carboxy-AA), or compiled from two sets of experiments (others). The raw data are listed in Supplementary Table 2.

FAs can also be subjected to CYP-mediated oxidation and metabolized to HETEs or epoxyeicosatrienoic acids (EETs). In this study, we monitored 22 species of CYP-produced metabolites and detected five species within the quantifiable range (Figure 6): 16-HETE and 20-carboxy-AA as AA metabolites; 9,10-dihydroxyoctadecenoic acid (9,10-DiHOME) and 12,13-DiHOME as LA metabolites; and 17,18-dihydroxyeicosatetraenoic acid (17,18-DiHETE) as an EPA metabolite. We found that the amount of ω-6 FA metabolites produced by CYP enzymes did not change after PNI (Figures 6A,B). In contrast, PNI markedly increased the levels of 17,18-DiHETE on day 7 (Figure 6C). Thus, we found that these CYP-produced metabolites in the DRG showed changes similar to LOX-produced metabolites after PNI.

### PNI affects the metabolism of lipids other than the biosynthetic products by COX/LOX/CYP enzymes

**Figure 7:**
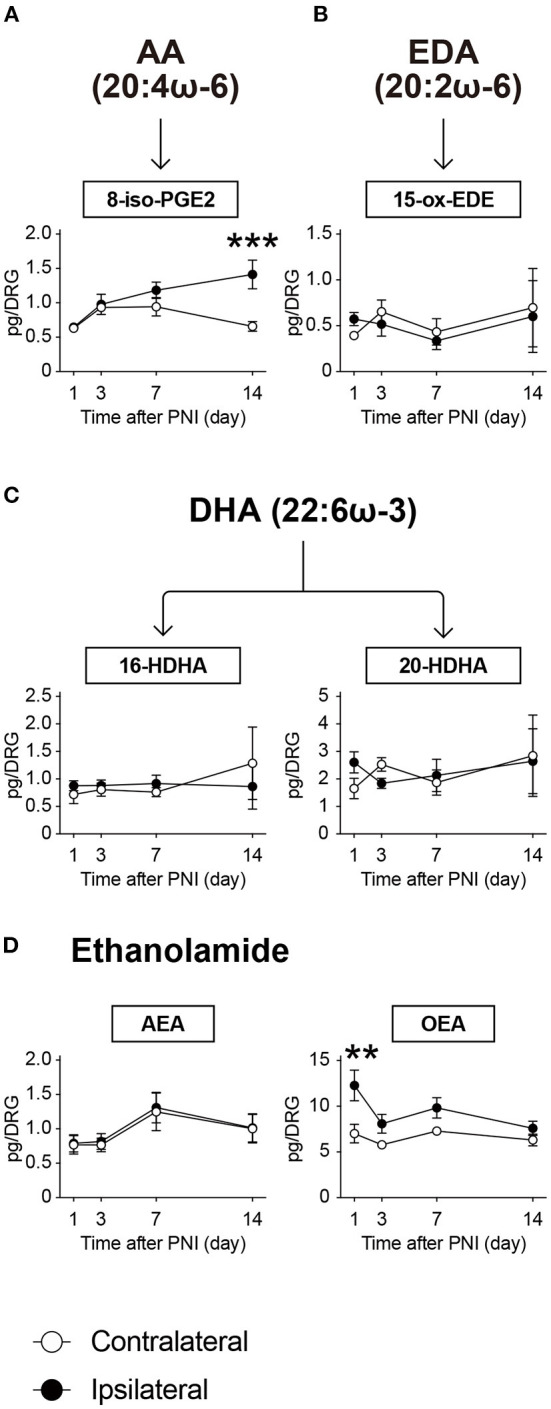
PNI affects the metabolism of isoprostane and ethanolamide in the DRG. The metabolic pathways of **(A)** arachidonic acid (AA), **(B)** eicosadienoic acid (EDA), **(C)** docosahexaenoic acid (DHA) after non-enzymatic oxidation, and **(D)** ethanolamide-related species. In total, six species could be quantified (*n* = 3–6; **P < 0.01, ***P < 0.001 vs. the contralateral side). Values are means ± s.e.m. The presented data are from one set of experiments (Day 1 of 8-iso-PGE_2_, Days 1, 3, and 14 of 15-ox-EDE, Day 1 of 16-HDHA, and Day 1 of 20-HDHA), or compiled from two sets of experiments (others). The raw data are listed in Supplementary Table 2.

Various endogenous lipid species are produced by non-enzymatic oxidation. In this study, we quantified four species (Figures 7A–C): 8-iso-PGE_2_ as an AA metabolite; 15-ox-EDE (also known as 15-KEDE) as a metabolite of eicosadienoic acid (20:2ω-6); and 16-HDHA and 20-HDHA as DHA metabolites. Among these, 8-iso-PGE_2_ level was significantly increased on day 14 after PNI (Figure 7A). However, the levels of the other three species were unaffected by nerve injury until day 14 (Figures 7B,C).

We also measured ethanolamide-related species and detected arachidonoylethanolamide (AEA) and oleoylethanolamide (OEA) within the calibration range. We found that OEA transiently increased on the day after PNI, whereas AEA was not affected (Figure 7D). These results suggest that PNI affects lipid metabolism, including the products of major metabolic enzymes as well as non-enzymatic products or ethanolamides in the DRG.

## Discussion

Lipids play crucial homeostatic roles in the healthy body and undergo qualitative and quantitative changes after injury and infection as well as in chronic diseases (26–28). Many studies have demonstrated that PNI alters the metabolism of FAs and other lipid classes, such as cholesterols, in the DRG, which are involved in pain and regeneration (14, 29–31). Although most previous studies have provided information regarding changes of particular lipid species or transcriptomic changes at a single time point, there is a lack of lipidomic information that can provide a comprehensive view of lipids in time dependent manner. Here, we showed how PNI alters FAs metabolism in the DRG on days 1, 3, 7, and 14.

Several groups have reported an increase in prostanoids, such as PGE_2_ and 6-keto-PGF_1α_, in the injured nerves (32–36); however little information is available regarding the DRG. Additionally, there are conflicting reports on changes in PGE_2_ levels after PNI, which either increased or remained unchanged (33, 35). In our study, most of the prostanoids and their derived species were significantly increased in the DRG at 14 days after PNI, regardless of whether they were derived from ω-6 or ω-3 FA; among them, PGD_1_, PGD_2_, TXB_2_, and 12-HHT were also increased on Day 7. COX-1 was reported to be expressed in small-diameter primary sensory neurons in the DRG, whereas COX-2 was not detected (37, 38). In contrast, non-neuronal cells were found to express both COX-1 and COX-2 (39). Furthermore, several recent studies using single-cell RNA sequencing (scRNA-seq) analysis have shown that terminal prostanoid synthases, such as PGD synthase, PGE synthase, and TXA synthase, are abundantly expressed in non-neuronal cells (5, 29, 40). These scRNA-seq resources have also shown that PNI upregulates the expression of terminal prostanoid synthases in macrophages, satellite glial cells (SGCs), endothelial cells, and fibroblasts in the DRG (5, 29). Given that various immune cells infiltrate, proliferate, and activate in response to nerve injury in the DRG (41, 42), the increased levels of prostanoids observed in this study might be of non-neuronal origin. However, we observed these increases only on days 7 and 14, despite the fact that the number of non-neuronal cells is increased and terminal PG synthases are upregulated by day 3 after nerve injury (5, 29, 43). Moreover, we observed the transient increase of free EPA levels after PNI, but not AA and DHA. As it was not consistent with the increasing time course of PGE_3_, the activity of metabolic enzymes may be more important in controlling prostanoid levels than the amounts of free FAs. These metabolic profiles could only be revealed by measuring the metabolites themselves, which are not encoded by genes; however, the related enzymes that regulate prostanoid levels, such as lysophospholipid acyltransferases (9), phospholipase A (44), and their catabolic enzymes, need to be evaluated.

We revealed that PNI induced differential metabolism of LOX-produced FA metabolites, compared with that of COX-produced metabolites. We found LTD_4_ to be an increased metabolite from ω-6 FA after PNI, whereas the others were unchanged. Increased LTD_4_ is thought to be derived from macrophages because both *Alox5* (5-LOX) and *Alox5ap* (arachidonate 5-lipoxygenase activating protein, FLAP) are selectively expressed in macrophages in the DRG (5, 29). Although LA-derived LOX-produced metabolites, 9- and 13-HODEs, have been reported to be increased in the DRG in the spared nerve injury model or in the chemotherapy-induced peripheral neuropathy model (45, 46), we did not observe quantitative changes in these metabolites during our study. This discrepancy might be due to the differences in nerve injury models (spared nerve injury or spinal nerve injury), but not due to the strain and age of mice (almost the same as this study). Intriguingly, we found that PNI transiently increased the levels of ω-3 FA-derived LOX-produced metabolites (15-HEPE, 14-HDHA, and 17-HDHA) in the DRG on days 3 and 7, and these were returned to the same level as the contralateral side by day 14. It is reported that 15-HEPE and 17-HDHA are precursors of lipoxin A_5_ and D-series resolvins, respectively (19, 20), even though we could not detect these in the present study. Lipoxins and resolvins are well-known SPM family species, and many studies have demonstrated the anti-inflammatory and analgesic properties of their pharmacological treatment (20–23). The present study raises the following new questions: how are ω-3 FAs selectively subjected to LOX-mediated oxidation and why are ω-3 FA-derived metabolites transiently increased in a specified period? Further studies are needed to resolve these important questions and understand the pathology of nerve injuries.

Following PNI, the amount of 8-iso-PGE_2_, which is synthesized by non-enzymatic oxidation, was significantly increased. An isoprostane, 8-iso-PGE_2_ is a biomarker of oxidative stress; thus, it is indicated that PNI elevates oxidative stress. In fact, several reports have demonstrated elevated oxidative stress after nerve injury (31, 47). Therefore, our results are consistent with the previous reports.

Our lipidomic analysis revealed that PNI also affects the amount of ethanolamides in the DRG. Among ethanolamide-related species, AEA is one of the most studied endocannabinoids and acts on type 1 cannabinoid receptors (48). In this study, although AEA levels were not altered, PNI increased the amount of OEA, which was reported to activate peroxisome proliferator-activated receptor-α (PPARα), with a median effective concentration of 0.12 μM (49). Recently, PPARα is reported to be enriched in SGCs in the DRG, and the expression of PPARα target genes is upregulated 3 days after nerve injury (29). Moreover, activation of PPARα signaling promotes axonal regeneration in peripheral nerves (29). Although further investigation is needed, the increased OEA may be an acute response to promote axonal regeneration after nerve injury.

We detected only five CYP-produced metabolites in our measurement. Although the metabolites derived from LA, such as epoxyoctadecenoic acids and DiHOMEs, have been shown to be increased in the DRG in several pain models, including the spared nerve injury model (46, 50, 51); however, in this study, the spinal nerve injury procedure did not elicit the changes in DiHOMEs from LA. In addition, as for EETs that could not be detected in the present study, detection sensitivity can be improved by using more suitable methods for measuring EETs, and further analysis may reveal interesting phenomena.

As important evidence, it has been reported that excessive dietary ω-6 FA induces damages to the peripheral nervous systems and results in sensory abnormalities (52). Furthermore, sciatic nerve transection increases AA-containing phosphatidylcholine in the mouse DRG (53). These reports and the evidence provided from the present study imply the importance of quality of lipid in our health and disease. Given that most of FAs are stored as glycerophospholipids, the studies focused on glycerophospholipids will be needed to understand PNI pathologies.

In conclusion, we presented the temporal changes in FA metabolism in the DRG after nerve injury. Moreover, we demonstrated the importance of focusing on metabolites themselves. It is becoming increasingly important to study biology through the lipidome or metabolome in combination with the transcriptome. However, because some FA metabolites and lipid species can be synthesized through several pathways, careful consideration should be used when focusing on such species. We expect that our findings will help identify novel principles of the pathology of nerve injury, contribute to pain alleviation, and promote nerve regeneration.

## Materials and methods

### Mice

C57BL/6 mice were purchased from CLEA Japan (Japan). All the mice used were 7–9 weeks old at the start of the experiments. Animals were housed in groups of 3–6 per cages with lights on from 8:00 to 20:00 and were provided food and water *ad libitum*. All animal experiments were conducted according to the guidelines of the Animal Research Committee of the National Center for Global Health and Medicine, using protocols approved by the committee.

### Peripheral nerve injury

We only used male mice for the spinal nerve injury procedure as a model of neuropathic pain (54). Briefly, mice were anesthetized with isoflurane and a small incision was made on the back. The L5 transverse process was then removed to expose the L4 spinal nerve. The exposed L4 spinal nerve was carefully cut, and then, the wound and skin were sutured with 5-0 silk.

### Behavioral test

Mechanical sensitivity was assessed using the von Frey test. Each mouse was placed in a wire mesh cage and habituated for more an hour before testing. Calibrated von Frey filaments (0.02–2.0 g) were applied to the mid-plantar skin of each hind paw. The 50% paw withdrawal threshold was determined using the up-down method (54).

### Sample preparation for lipid measurement

Mice were deeply anesthetized and perfused with ice-cold saline through the left ventricle. The L4 DRG were quickly removed and frozen in liquid nitrogen. Frozen tissues were pulverized and the lipid components were extracted for 60 min at 4 °C in methanol spiked with a deuterium-labeled internal standard mixture, which included PGD_2_-d4, PGE_2_-d4, PGF_2α_-d9, 6-keto-PGF_1α_-d4, TXB_2_-d4, LTB_4_-d4, LTC_4_-d5, LTD_4_-d5, LTE_4_-d5, 5-HETE-d8, 12-HETE-d8, 15-HETE-d8, AEA-d4, OEA-d4, tetranor-PGEM-d6, AA-d8, EPA-d5, and DHA-d5. The supernatants were collected after centrifugation at 15,000 × *g* for 10 min at 4 °C. The methanol extracts were purified by a solid-phase method using an Oasis HLB column (Waters).

### Lipid measurement

Endogenous FA metabolites were measured using a triple quadrupole mass spectrometer LCMS-8060 (Shimadzu) as previously described (25, 55). Briefly, a reverse-phase column (Kinetex C8, 2.1 × 150 mm, 2.6 μm, Phenomenex) was used for chromatographic separation with a binary mobile phase comprising 0.1% formic acid/water (mobile phase A) and acetonitrile (mobile phase B). The gradient of the mobile phase (%A/%B) was programmed as follows: 0 min (90/10), 5 min (75/25), 10 min (65/35), 20 min (25/75), 20.1–28 min (5/95), 28.1–30 min (90/10). The flow rate was 0.4 ml/min, and the column temperature was 40 °C. The selected reaction-monitoring transitions are listed in Supplementary Table 1. Raw data were analyzed by using LabSolutions Insight (Shimadzu). The signals were compared with the standard curves for quantification. Although each sample containing six (respectively 8) DRGs was analyzed using LC-MS, the graphs show the values of metabolites calculated per one DRG.

### Statistical analyses

Data were analyzed using two-way ANOVA with a *post-hoc* Bonferroni test using GraphPad Prism 7 software to determine differences among groups. Statistical significance was set at *P* < 0.05.

## Data availability statement

The original contributions presented in the study are included in the article/Supplementary materials, further inquiries can be directed to the corresponding authors.

## Ethics statement

The animal study was reviewed and approved by the Animal Research Committee of the National Center for Global Health and Medicine.

## Author contributions

SY designed and performed most of the experiments, analyzed the data, and wrote the manuscript. TH-Y performed LC-MS analysis and analyzed the data. TS supervised the study. HS conceived the study, supervised the overall project, and wrote the manuscript. All the authors have read, discussed the manuscript, and contributed to the article and approved the submitted version.

## Funding

This work was supported by JSPS KAKENHI Grant Numbers JP19K16938 and JP21K15309 (to SY), the Core Research for Evolutional Science and Technology (CREST) from the Japan Agency for Medical Research and Development (AMED) under Grant Number 22gm0910011 (to HS), the Project for Cancer Research and Therapeutic Evolution (P-CREATE) from AMED under Grant Number 21cm0106116 (to HS), by The Nakatomi Foundation (to SY), and by Takeda Science Foundation 15668360 (to TS). SY was a research fellow of the JSPS (21J00759).

## Conflict of interest

The authors declare that the research was conducted in the absence of any commercial or financial relationships that could be construed as a potential conflict of interest.

The Department of Lipid Signaling and Lipid Life Science, National Center for Global Health and Medicine, is financially supported by Ono Pharmaceutical Co., Ltd.

## Publisher's note

All claims expressed in this article are solely those of the authors and do not necessarily represent those of their affiliated organizations, or those of the publisher, the editors and the reviewers. Any product that may be evaluated in this article, or claim that may be made by its manufacturer, is not guaranteed or endorsed by the publisher.

## Abbreviations

AA: arachidonic acid
AEA: arachidonoyl ethanolamide
COX: cyclooxygenase
CYP: cytochrome-P_450_
DHA: docosahexaenoic acid
DiHETE: dihydroxyeicosatetraenoic acid
DiHOME: dihydroxyoctadecenoic acid
DRG: dorsal root ganglion
EET: epoxyeicosatrienoic acid
EPA: eicosapentaenoic acid
FA: fatty acid
HDHA: hydroxydocosahexaenoic acid
HEPE: hydroxyeicosapentaenoic acid
HETE: hydroxyeicosatetraenoic acid
HODE: hydroxyoctadecadienoic acid
HpETE: hydroperoxyeicosatetraenoic acid
HpODE: hydroperoxyoctadecadienoic acid
LA: linoleic acid
LC-MS/MS: liquid chromatography-mass spectrometry
LOX: lipoxygenase
LT: leukotriene
OEA: oleoyl ethanolamide
PG: prostaglandin
PNI: peripheral nerve injury
PPARα: peroxisome proliferator-activated receptor-α
scRNA-seq: single cell RNA-sequencing
SGC: satellite glial cell
SPM: specialized pro-resolving lipid mediator
TRPV1: transient receptor potential vanilloid 1
TX: thromboxane
12-HHT: 12-hydroxyheptadecatrienoic acid
15-HETrE: 15-hydroxytrienoic acid.
