# Supplementary material for: Profiling of fatty acid metabolism in the dorsal root ganglion after peripheral nerve injury
Source: Front Pain Res (Lausanne). 2022 Jul 29;3:948689. doi: 10.3389/fpain.2022.948689 (PMC9372306; doi:10.3389/fpain.2022.948689)
Supplement: Supplementary file 1 [file Image_1.pdf]

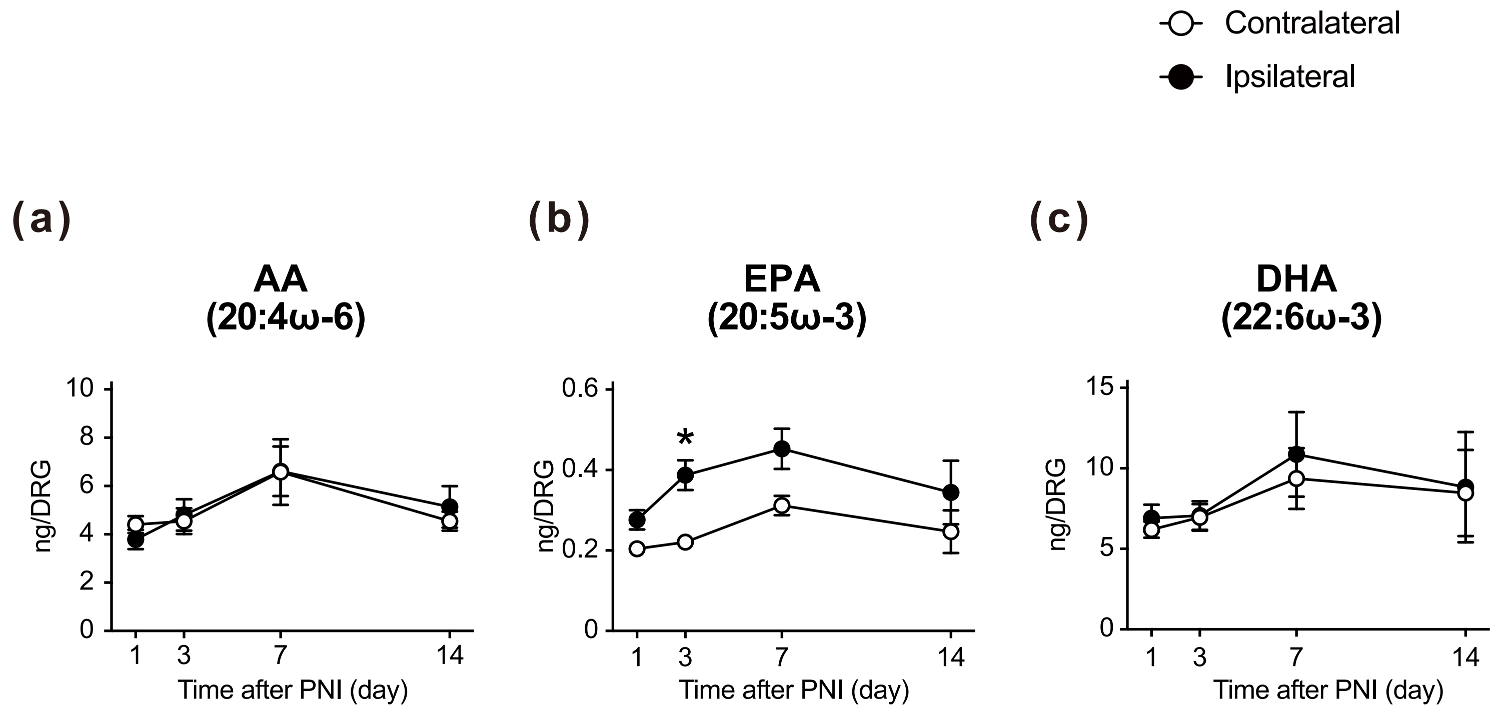

Supplementary Figure 1: The levels of free fatty acid after PNI.

The amount of (a) arachidonic acid (AA), (b) eicosapentaenoic acid (EPA), and (c) docosahexaenoic acid (DHA) in the DRG. (n = 3–6; *P < 0.05 *vs.* the contralateral side). Values are means ± s.e.m. The presented data are from one set of experiments (AA), or compiled from two sets of experiments (EPA, DHA). The raw data are listed in Supplementary Table 2.
